# Perforated marginal ulcer after Roux-en-Y gastric bypass: a national analysis of operative approaches, outcomes, and practice variations, 2018–2022

**DOI:** 10.3389/fsurg.2026.1907935

**Published:** 2026-10-21

**Authors:** Wael Alkattan

**Affiliations:** College of Medicine, Alfaisal University, Riyadh, Saudi Arabia

**Keywords:** bariatric surgery, gastrojejunal ulcer, laparoscopic surgery, marginal ulcer, national inpatient sample, omental patch, perforation, Roux-en-Y gastric bypass

## Abstract

**Background:**

Perforated marginal (gastrojejunal) ulcer is an uncommon but life-threatening complication after Roux-en-Y gastric bypass. Evidence is limited to single-center series and one 2022 systematic review of approximately 600 patients; contemporary national International Classification of Diseases, Tenth Revision (ICD-10)-era data are lacking.

**Methods:**

We queried the National Inpatient Sample (2018–2022) for adults with a perforated gastrojejunal ulcer and a code indicating prior bariatric surgery. Operative approach was classified as laparoscopic patch, open patch, revisional gastrojejunostomy/reversal, or non-operative management. Survey-weighted statistics, negative-binomial (length of stay), gamma (total charges), and logistic (predictors of operative repair) models were estimated with hospital cluster-robust standard errors.

**Results:**

The cohort included 1,669 unweighted admissions (8,345 weighted). Operative repair was performed in 28.9% (laparoscopic patch 12.6%, open patch 7.2%, revisional/reversal 9.2%); 71.1% were managed non-operatively. Crude in-hospital mortality was 1.1% overall. Non-operative management rose from 64.1% (2018) to 72.0% (2022), while laparoscopic patch declined from 18.1% to 12.1%. Open patch was associated with longer length of stay than laparoscopic patch [incidence-rate ratio 1.41, 95% confidence interval (CI) 1.13–1.77] and higher complication rates after correction for multiple comparisons of sepsis, acute kidney injury, respiratory failure, and venous thromboembolism. Elective admission strongly predicted operative repair (adjusted odds ratio 2.58, 95% CI 1.74–3.83), elective admissions were concentrated in the revisional group, but the reason for elective admission could not be determined from administrative data. Operative rates declined modestly each year (adjusted odds ratio 0.91, 95% CI 0.84–0.98). Case mix differed between laparoscopic-first and open-first centers.

**Conclusions:**

In the largest contemporary national cohort to date, non-operative management dominated and increased during 2018–2022. Among operatively managed patients, laparoscopic patch repair was associated with shorter length of stay and a more favorable complication profile than open repair. Because operative approach was not randomized and patients selected for open repair may have carried greater physiologic severity or technical complexity, these associations support—but do not establish—a laparoscopic-first approach where anatomy and physiology permit. Prospective evidence is needed to refine operative vs. non-operative triage.

## 1 Introduction

Severe obesity affects approximately 9% of US adults (1), and Roux-en-Y gastric bypass (RYGB) remains a durable, mortality-reducing operation for patients with morbid obesity and metabolic disease (2, 3). Marginal ulceration at the gastrojejunal anastomosis is one of the most common late complications of RYGB, with a reported incidence of approximately 1%–16% across single-center and pooled series (4, 5). While most marginal ulcers respond to acid suppression and modification of risk factors, a small but clinically consequential subset perforate and present as surgical emergencies (4, 6).

The pathogenesis of marginal ulcer is multifactorial. Local factors include anastomotic ischemia, exposure of the jejunal mucosa to acid produced by a small or fistulized gastric pouch, and foreign material such as non-absorbable suture or staples (4, 7). Modifiable systemic factors include tobacco smoking (8), chronic non-steroidal anti-inflammatory drug (NSAID) exposure (9), untreated Helicobacter pylori infection (10, 11), diabetes, alcohol use, and incomplete acid-suppressive therapy—further complicated by reduced absorption of intact proton pump inhibitor capsules in the postbypass anatomy (12, 13). Two recent meta-analyses have systematically synthesized these risk factors (10, 12).

Despite a robust body of literature on uncomplicated marginal ulcer, the perforated phenotype has been underexamined. The most comprehensive synthesis to date is a 2022 systematic review that pooled approximately 600 patients across all available reports (6). Large administrative and registry databases have addressed marginal ulcer more broadly: Analyses of the Metabolic and Bariatric Surgery Accreditation and Quality Improvement Program (MBSAQIP) have characterized early (30-day) marginal ulceration after RYGB (14, 15), and an International Classification of Diseases (ICD)-9-era National Inpatient Sample analysis examined Helicobacter pylori as a predictor of marginal ulceration after bariatric surgery (16). None of these studies, however, examined the perforated phenotype, its operative management, or its inpatient outcomes. Single-center series describing operative repair have been small (typically 22–66 patients) (17–19), and contemporary national data in the ICD-10 era are absent. The relative roles of laparoscopic patch repair, open patch repair, revisional gastrojejunostomy, and non-operative management at a population level—and how those choices have evolved during the 5-year study period—have not been characterized.

We therefore queried the Healthcare Cost and Utilization Project (HCUP) National Inpatient Sample (NIS) from 2018 through 2022 to provide the first national, contemporary description of perforated marginal ulcer after RYGB. Our specific aims were (i) to estimate the national annual volume of admissions and characterize the patient population; (ii) to describe the distribution of operative approaches and to test whether utilization changed over the 5-year period; (iii) to compare in-hospital mortality, complication burden, length of stay, and total charges across operative approaches; and (iv) to explore center-level practice variations by stratifying admissions according to the dominant patch approach (laparoscopic first vs. open first) at the treating hospital.

## 2 Materials and methods

### 2.1 Data source

The Healthcare Cost and Utilization Project NIS is the largest publicly available, all-payer inpatient care database in the United States, sponsored by the Agency for Healthcare Research and Quality. The NIS uses a stratified single-stage cluster sampling design that captures a 20% sample of US community-hospital discharges and provides discharge-level survey weights for national projections (20). We pooled five sequential annual files (2018–2022).

### 2.2 Cohort definition

We identified adult admissions (age ≥ 18 years) that carried both a diagnosis of acute or chronic gastrojejunal ulcer with perforation—with or without hemorrhage, in any diagnosis position—and a status code indicating prior bariatric surgery. Pregnancy admissions and trauma admissions were excluded, as were records missing the survey weight or the in-hospital mortality flag. A prespecified sensitivity cohort additionally included admissions captured by postprocedural complication codes for gastrointestinal disorders. Specific ICD-10-CM codes used for case ascertainment are listed in the Supplementary Appendix S2.

### 2.3 Operative approach

Operative approach was assigned from inpatient procedure codes into four mutually exclusive groups: laparoscopic patch repair, open patch repair, revisional gastrojejunostomy or reversal of the bypass, and non-operative management. Patch repair encompassed suture or omental repair of the stomach or jejunum; revisional repair encompassed takedown of the gastrojejunal anastomosis or reversal of the bypass. Diagnostic laparoscopy without therapeutic intervention was considered non-operative for the primary analysis. To explore center-level practice variation, hospitals were classified as laparoscopic-first or open-first based on the dominant patch approach across their patch admissions during the study period.

### 2.4 Outcomes

The primary outcome was in-hospital mortality. Secondary outcomes included length of stay, total charges (inflation-adjusted to 2022 US dollars), and a panel of in-hospital complications: sepsis, acute kidney injury, respiratory failure, venous thromboembolism, surgical site infection, anastomotic leak, packed red blood cell transfusion, and postprocedural gastrointestinal complications. Diagnosis-based outcomes were identified using diagnosis codes, and transfusion was identified using procedure codes; the definitions are provided in Supplementary Appendix S2.

### 2.5 Covariates

Patient-level covariates included age, sex, race/ethnicity, primary expected payer, neighborhood income (ZIP code quartile of median household income), weekend admission, and elective admission status. Hospital-level covariates included census region and teaching/location status (rural, urban non-teaching, urban teaching). Comorbidity burden was summarized using the Elixhauser comorbidity count (21) and the van Walraven weighted comorbidity score (22), both computed from diagnosis codes using the Quan algorithms (23). Behavioral exposures of interest included tobacco use, non-steroidal anti-inflammatory drug exposure, Helicobacter pylori infection, obesity diagnosis, and substance use; these were captured from standard ICD-10-CM codes (full list in the Supplementary Appendix S2).

### 2.6 Statistical analysis

All analyses applied the NIS discharge weight and accounted for the stratified cluster design with cluster-robust standard errors at the hospital level. Continuous variables are summarized as mean (standard deviation) or median (interquartile range), and categorical variables as counts and weighted percentages. Group comparisons used survey-weighted chi-square tests for proportions and Kruskal–Wallis tests for means; standardized mean differences are also reported.

Length of stay was modeled with negative-binomial regression, and total charges with a gamma generalized linear model with a log link, both adjusted for operative approach, age, sex, comorbidity burden, calendar year (centered at 2020), and elective admission status. Logistic regression for receipt of any operative repair was estimated on the same covariates plus modifiable exposures of interest (smoking, non-steroidal anti-inflammatory drug exposure, *Helicobacter pylori*, obesity, and substance use). The Benjamini–Hochberg false discovery rate (FDR) procedure was applied to the modifiable-exposure panel. Mortality was reported descriptively with Wilson confidence intervals because the small number of in-hospital deaths precluded a stable multivariable model.

Prespecified sensitivity analyses excluded the COVID-19 year (2020), excluded 2018 to address residual coding transition concerns, restricted the cohort by removing admissions identified only through postprocedural complication codes, and re-estimated the operative-approach contrasts using inverse-probability-of-treatment weighting and overlap weights (24) for the laparoscopic-vs.-open patch comparison. A leave-one-out sensitivity analysis classified hospital practice using other patch-repair admissions within the same hospital and calendar year. Sparse data and model separation prevented reliable estimation of the adjusted model (Supplementary Appendix S3). Two-sided *p* < 0.05 was considered statistically significant. Analyses were performed in Python 3.11 with the statsmodels and pyreadstat libraries.

### 2.7 Ethics statement

This study used a deidentified, publicly available database; institutional review board approval was not required.

## 3 Results

### 3.1 Cohort assembly

After applying the exclusion criteria, the primary analysis included 1,669 unweighted admissions, corresponding to 8,345 nationally weighted admissions (Figure 1). The mutually exclusive operative groups were laparoscopic patch (*n* = 210), open patch (*n* = 120), revisional gastrojejunostomy or reversal (*n* = 153), and non-operative management (*n* = 1,186). The sensitivity cohort, which additionally included admissions identified through postprocedural gastrointestinal complication codes, contained 10,552 admissions.

**Figure 1:**
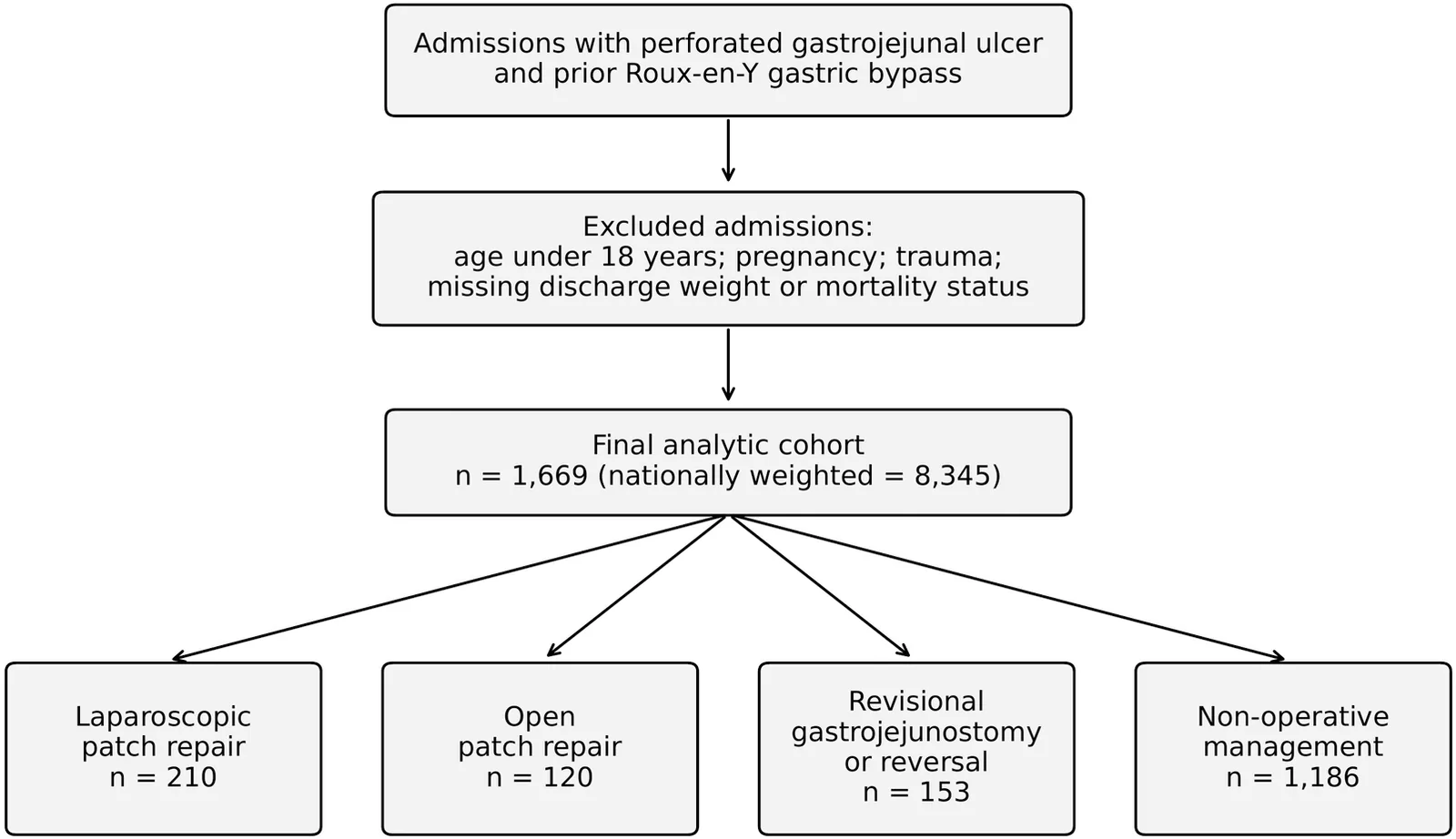
Cohort assembly. Admissions with perforated gastrojejunal ulcer and prior bariatric surgery were identified in the National Inpatient Sample, 2018–2022. After applying the exclusion criteria, the analytic cohort included 1,669 unweighted admissions, representing 8,345 nationally weighted admissions. The four treatment groups are mutually exclusive under the study classification algorithm.

### 3.2 Baseline characteristics by operative approach

Patient-level demographics were broadly similar across approaches (Table 1). Mean age ranged from 51.1 years (laparoscopic patch) to 54.0 years (open patch), and women constituted 80%–85% of each group. Smoking prevalence was highest in the open patch group (35.0%) compared with 23.3% in laparoscopic patch and 27.0% across the cohort. NSAID exposure and *Helicobacter pylori* coding were uncommon. Comorbidity burden by Elixhauser count was similar across groups (mean 3.8–4.2). Most admissions in each group were managed at urban teaching hospitals (61.7%–81.1% across groups), with the lowest proportion in the open patch group.

**Table 1:** Baseline characteristics by operative approach.

Variable	Laparoscopic patch (*n* = 210; w*N* = 1,050)	Open patch (*n* = 120; w*N* = 600)	Revisional gastrojejunostomy (*n* = 153; w*N* = 765)	Non-operative (*n* = 1,186; w*N* = 5,930)	*p*-Value	Maximum SMD
Demographics						
Age, years, mean (SD)	51.1 (13.2)	54.0 (11.9)	53.7 (11.9)	52.4 (12.7)	0.192	0.135
Female, *n* (%)	**173** **(82.4)**	**102** **(85.0)**	**124** **(81.0)**	**953** **(80.4)**	**0** **.** **027**	**0** **.** **123**
Admission						
Weekend admission, *n* (%)	**70** **(33.3)**	**28** **(23.3)**	**35** **(22.9)**	**296** **(25.0)**	**<0** **.** **001**	**0** **.** **185**
Elective admission, *n* (%)	**13** **(6.2)**	Suppressed	**40** **(26.1)**	**58** **(4.9)**	**<0** **.** **001**	**0** **.** **614**
Behavioral/predictors of interest						
Smoking, *n* (%)	**49** (**23.3)**	**42** (**35.0)**	**36** (**23.5)**	**320** (**27.0)**	**<0**.**001**	**0**.**174**
NSAID exposure, *n* (%)	Suppressed	Suppressed	Suppressed	**41** (**3.5)**	**<0**.**001**	**0**.**141**
*H. pylori*, *n* (%)	Suppressed	Suppressed	Suppressed	Suppressed	**0**.**003**	**0**.**124**
Obesity/BMI diagnosis, *n* (%)	**100** (**47.6)**	**44** (**36.7)**	**77** (**50.3)**	**485** (**40.9)**	**<0**.**001**	**0**.**19**
Substance use, *n* (%)	27 (12.9)	16 (13.3)	19 (12.4)	129 (10.9)	0.079	0.075
Comorbidities						
Elixhauser count, mean (SD)	3.8 (2.0)	3.9 (1.9)	4.2 (2.3)	3.8 (2.0)	0.097	0.232
Van Walraven score, mean (SD)	2.1 (6.5)	3.5 (7.8)	3.5 (8.6)	2.8 (7.3)	0.432	0.105
Diabetes Uncomplicated, *n* (%)	**24** **(11.4)**	**12** **(10.0)**	**24** **(15.7)**	**162** **(13.7)**	**0** **.** **004**	**0** **.** **113**
Diabetes Complicated, *n* (%)	**26** **(12.4)**	Suppressed	**16** **(10.5)**	**122** **(10.3)**	**<0** **.** **001**	**0** **.** **164**
HTN uncomplicated, *n* (%)	72 (34.3)	45 (37.5)	56 (36.6)	458 (38.6)	0.053	0.09
HTN complicated, *n* (%)	**21** **(10.0)**	**14** **(11.7)**	**20** **(13.1)**	**103** **(8.7)**	**<0** **.** **001**	**0** **.** **141**
Chronic pulmonary, *n* (%)	**45** **(21.4)**	**19** **(15.8)**	**38** **(24.8)**	**212** **(17.9)**	**<0** **.** **001**	**0** **.** **17**
Renal failure, *n* (%)	**14** **(6.7)**	Suppressed	**11** **(7.2)**	**65** **(5.5)**	**0** **.** **010**	**0** **.** **113**
CHF, *n* (%)	12 (5.7)	Suppressed	Suppressed	67 (5.6)	0.306	0.069
Liver, *n* (%)	13 (6.2)	Suppressed	11 (7.2)	70 (5.9)	0.130	0.079
Depression, *n* (%)	**53** **(25.2)**	**26** **(21.7)**	**45** **(29.4)**	**288** **(24.3)**	**0** **.** **005**	**0** **.** **116**
Alcohol, *n* (%)	**18** **(8.6)**	Suppressed	Suppressed	**74** **(6.2)**	**<0** **.** **001**	**0** **.** **093**
Hospital						
Rural, *n* (%)	Suppressed	18 (15.0)	Suppressed	56 (4.7)	<0.001	0.350
Urban non-teaching, *n* (%)	Suppressed	**28** **(23.3)**	Suppressed	**168** **(14.2)**	**<0** **.** **001**	**0** **.** **237**
Urban teaching, *n* (%)	**167** **(79.5)**	**74** **(61.7)**	**119** **(77.8)**	**962** **(81.1)**	**<0** **.** **001**	**0** **.** **441**

Maximum SMD = maximum pairwise SMD vs. the largest group. Weighted percentages using discharge weight. *p*-Values from weighted chi-square (categorical) or Kruskal–Wallis tests (continuous). Operative-approach strata are mutually exclusive, with priority Revisional gastrojejunostomy > Patch (Open patch/Laparoscopic patch) > Non-operative. Elective admission is retained for completeness: Elective admissions clustered in the revisional gastrojejunostomy/reversal group (26.1%). Elective admissions were concentrated in the revisional group, but the reason for elective admission could not be determined from administrative data. Values are suppressed to protect confidentiality, including corresponding percentages and additional cells needed to prevent calculation of suppressed counts. Comparison statistics for affected rows are also withheld. Bold values indicate statistical significance (*p* < 0.05). CHF, congestive heart failure; HTN, hypertension; NSAID, non-steroidal anti-inflammatory drug; SMD, standardized mean difference.

### 3.3 Operative management and 5-year trends

Across the cohort, 28.9% of admissions received operative repair; 21.0% had a patch (13.2% laparoscopic, 7.8% open), 9.2% underwent revisional gastrojejunostomy or RYGB reversal, and 1.2% had diagnostic laparoscopy without therapeutic intervention (Table 2). Non-operative management rose from 64.1% in 2018 to a peak of 74.7% in 2020 and remained at 72.0% in 2022, while laparoscopic patch repair declined from 18.1% in 2018 to 12.1% in 2022 (Figure 2). Open patch and revisional repair were stable at 6%–9% per year.

**Table 2:** Operative management of perforated marginal ulcer after RYGB, 2018–2022.

Indicator	Unweighted *N*	Weighted (%)	Weighted *N* (event)
Any operative repair	483	28.9	2,415
Repair procedure (any included approach)	351	21.0	1,755
Revisional/takedown of GJ	153	9.2	765
Diagnostic laparoscopy	20	1.2	100

Rates weighted (discharge weight). Cells with unweighted *n* < 11 suppressed per HCUP data-use policy. Counts for Patch (lap/open) and Revisional GJ are NOT mutually exclusive: A case with both a patch and a revisional procedure code appears in both rows here, but is assigned to Revisional gastrojejunostomy in Table 1 under the priority Revisional > Patch > Non-operative. Diagnostic laparoscopy cases without patch or revisional codes are classified as Non-operative in Tables 1, 3. GJ, gastrojejunostomy; HCUP, Healthcare Cost and Utilization Project. Procedure categories may overlap and do not form an exhaustive mutually exclusive breakdown.

**Figure 2:**
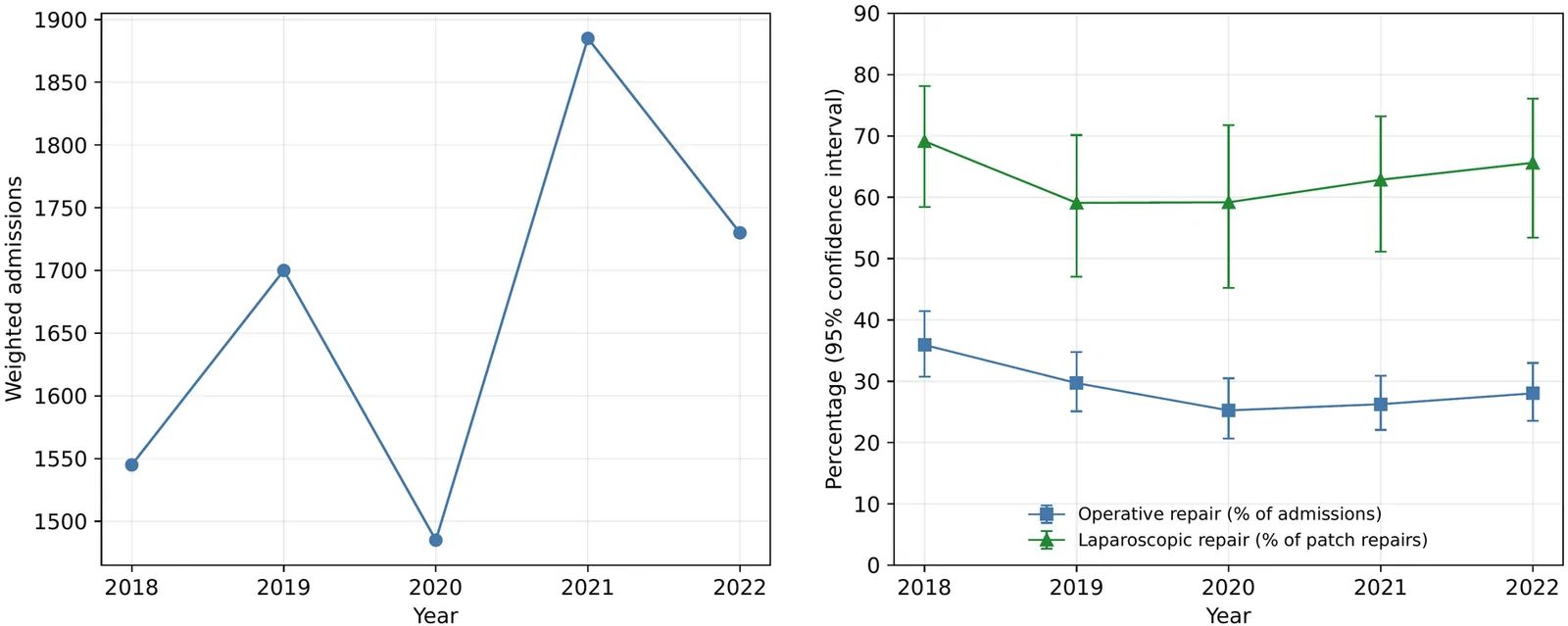
Annual admission volume and treatment patterns, 2018–2022. The left panel shows nationally weighted admissions by calendar year. The right panel shows operative repair as a percentage of all included admissions and laparoscopic repair as a percentage of admissions classified as laparoscopic or open patch repair. Error bars represent descriptive Wilson 95% confidence intervals calculated from unweighted counts, not confidence intervals adjusted for the full survey design. Mortality is not displayed.

### 3.4 In-hospital mortality

Crude in-hospital mortality was 1.1% across the full cohort, corresponding to 19 unweighted deaths and approximately 95 weighted deaths. Approach-specific mortality estimates were suppressed to protect confidentiality. The small number of deaths precluded a stable multivariable mortality model.

### 3.5 Complications

After false discovery rate adjustment, open patch repair was associated with significantly higher rates of sepsis (20.0% vs. 14.3%; *q* = 0.008), acute kidney injury (20.0% vs. 9.5%; *q* < 0.001), compared with laparoscopic patch repair (Tables 3, 4). Postprocedural gastrointestinal complications were also more common after open patch (14.2% vs. 10.5%; *q* = 0.050).

**Table 3:** In-hospital outcomes by operative approach.

Group	*N* (unw)	Weighted *N*	In-hospital mortality, *n* (%)	LOS (days), median (IQR)	Total charges (USD), median (IQR)	Sepsis, *n* (%)	AKI, *n* (%)	Respiratory failure, *n* (%)	VTE, *n* (%)	SSI, *n* (%)	Anastomotic leak, *n* (%)	Transfusion, *n* (%)	Postprocedural complication, *n* (%)
All PMU (full cohort)	1,669	8,345	19 (1.1)	5 (3–7)	62,848 (38,939–102,993)	240 (14.4)	162 (9.7)	Suppressed	Suppressed	Suppressed	Suppressed	Suppressed	156 (9.3)
Operative (any approach)	483	2,415	Suppressed	5 (4–8)	71,984 (46,555–121,761)	82 (17.0)	61 (12.6)	Suppressed	Suppressed	Suppressed	Suppressed	Suppressed	64 (13.3)
Laparoscopic patch	210	1,050	Suppressed	5 (3–7)	60,560 (40,975–103,835)	30 (14.3)	20 (9.5)	Suppressed	Suppressed	Suppressed	Suppressed	13 (6.2)	22 (10.5)
Open patch	120	600	Suppressed	6 (5–8)	70,961 (48,883–125,589)	24 (20.0)	24 (20.0)	12 (10.0)	Suppressed	Suppressed	Suppressed	Suppressed	17 (14.2)
Revisional gastrojejunostomy	153	765	Suppressed	5 (3–9)	81,674 (55,820–154,313)	28 (18.3)	17 (11.1)	14 (9.2)	Suppressed	Suppressed	Suppressed	16 (10.5)	25 (16.3)
Non-operative	1,186	5,930	Suppressed	5 (3–7)	59,181 (35,333**-**97,662)	158 (13.3)	101 (8.5)	58 (4.9)	13 (1.1)	23 (1.9)	Suppressed	75 (6.3)	92 (7.8)

VTE rate may reflect admission diagnoses; interpret with caution. Postprocedural complication captures postprocedural perforation/leak. Approach strata mutually exclusive (priority Revisional gastrojejunostomy > Patch > Non-operative). Values are suppressed to protect confidentiality, including corresponding percentages and additional cells needed to prevent calculation of suppressed counts. Comparison statistics for affected rows are also withheld. AKI, acute kidney injury; LOS, length of stay; SSI, surgical site infection; VTE, venous thromboembolism.

**Table 4:** FDR-adjusted complication comparison: laparoscopic vs. open patch repair.

Complication	Lap-patch (%)	Open patch (%)	SMD	P_raw	P_FDR
Sepsis	**14**.**29**	**20**.**0**	**0**.**152**	**0**.**003**	**0**.**008**
AKI	**9**.**52**	**20**.**0**	**0**.**299**	**<0**.**001**	**<0**.**001**
Respiratory failure	Suppressed	**10**.**0**	**0**.**32**	**<0**.**001**	**<0**.**001**
VTE	Suppressed	Suppressed	**0**.**119**	**0**.**023**	**0**.**047**
SSI	Suppressed	Suppressed	0.019	0.864	0.987
Anastomotic leak	Suppressed	Suppressed	0.0	1.000	1.000
Transfusion	6.19	Suppressed	0.052	0.356	0.474
Postprocedural complication	10.48	14.17	0.112	0.031	0.050

Weighted chi-square. P_FDR = Benjamini–Hochberg corrected. Bold values indicate statistical significance after Benjamini-Hochberg false-discovery-rate correction (*q* < 0.05). AKI, acute kidney injury; FDR, false discovery rate; SSI, surgical site infection.

### 3.6 Length of stay and charges

Median length of stay was 5 days (interquartile range 3–7) in the full cohort and 6 days (5–8) in the open patch group. In a negative-binomial model with laparoscopic patch as reference and adjustment for age, sex, comorbidity burden, calendar year, and elective status, open patch repair was associated with a 41% longer stay (incidence rate ratio 1.41, 95% CI 1.13–1.77; *p* = 0.002) (Tables 5, 6). Revisional repair and non-operative management did not differ significantly from laparoscopic patch in length of stay. In a gamma model for total charges, non-operative management was associated with significantly lower charges than laparoscopic patch (charge ratio 0.83, 95% CI 0.73–0.94; *p* = 0.005), while open patch and revisional repair were associated with non-significantly higher charges (ratios 1.22 and 1.16, respectively).

**Table 5:** Multivariable model for length of stay (negative-binomial regression).

Variable	Ratio (95% CI)	*p*-Value
Intercept	**4.21** (**3.47**–**5.09)**	**<0**.**001**
Non-operative (vs. laparoscopic patch)	0.99 (0.89–1.10)	0.831
Open patch (vs. laparoscopic patch)	**1.41** (**1.13**–**1.77)**	**0**.**002**
Revisional/takedown of GJ (vs. laparoscopic patch)	1.01 (0.87–1.18)	0.894
Age (per year)	**1.00** (**1.00**–**1.01)**	**0**.**002**
Female sex	1.06 (0.96–1.16)	0.235
Comorbidity score (Van Walraven)	**1.03** (**1.03**–**1.04)**	**<0**.**001**
Calendar year (centered at 2020)	0.98 (0.96–1.01)	0.152
Elective admission	0.98 (0.81–1.19)	0.846

Reference category: laparoscopic patch. Survey-weighted; hospital identifier cluster SEs. Elective admission retained here (no separation in continuous outcomes). Bold values indicate statistical significance (*p* < 0.05).

**Table 6:** Multivariable model for total hospital charges (gamma generalized linear model with log link).

Variable	Ratio (95% CI)	*p*-Value
Intercept	**77440.00** (**62050.00**–**96630.00)**	**<0**.**001**
Non-operative (vs. laparoscopic patch)	**0.83** (**0.73**–**0.94)**	**0**.**005**
Open patch (vs. laparoscopic patch)	1.22 (0.95–1.56)	0.118
Revisional/takedown of GJ (vs. laparoscopic patch)	1.16 (0.98–1.38)	0.088
Age (per year)	**1.00** (**1.00**–**1.01)**	**0**.**032**
Female sex	0.98 (0.89–1.09)	0.763
Comorbidity score (Van Walraven)	**1.03** (**1.03**–**1.04)**	**<0**.**001**
Calendar year (centered at 2020)	1.00 (0.97–1.03)	0.871
Elective admission	1.12 (0.91–1.37)	0.281

Reference category: laparoscopic patch. Survey-weighted; hospital identifier cluster SEs. Charges deflated to 2022 USD with BLS CPI-U. Elective admission retained here (no separation in continuous outcomes). Bold values indicate statistical significance (*p* < 0.05). CI, confidence interval.

### 3.7 Predictors of operative repair

In a multivariable logistic model, elective admission was the strongest predictor of receiving any operative repair (adjusted odds ratio 2.58, 95% CI 1.74–3.83; *p* < 0.001), reflecting that preplanned encounters in this cohort largely represent revisional procedures (Table 7). Elective admissions were concentrated in the revisional gastrojejunostomy/reversal group (26.1%; Table 1). Calendar year (centered at 2020) was associated with a modest year-over-year decline in operative repair (adjusted odds ratio 0.91 per year, 95% CI 0.84–0.98; *p* = 0.020). Among modifiable exposures of interest, the association before FDR correction of NSAID exposure with lower operative-repair odds (raw *p* = 0.045) did not survive false discovery rate adjustment (*q* = 0.142); smoking, *Helicobacter pylori*, obesity coding, and substance use were not associated with operative receipt.

**Table 7:** Multivariable predictors of receiving any operative repair (logistic regression).

Variable	aOR (95% CI)	*p*-Value
Intercept	**0.24** (**0.14**–**0.44)**	**<0**.**001**
Smoking	0.99 (0.78–1.27)	0.956
NSAID exposure	**0.45** (**0.21**–**0.98)**	**0**.**045**
*H. pylori* infection	0.93 (0.24–3.52)	0.912
Obesity/BMI diagnosis	1.22 (0.98–1.52)	0.080
Substance use	1.34 (0.96–1.88)	0.085
Age (per year)	1.00 (0.99–1.01)	0.467
Female sex	1.22 (0.92–1.61)	0.163
Comorbidity score (Van Walraven)	1.00 (0.99–1.02)	0.841
Calendar year (centered at 2020)	**0.91** (**0.84**–**0.98)**	**0**.**020**
Elective admission	**2.58** (**1.74**–**3.83)**	**<0**.**001**

Displayed *p*-values are before correction for multiple comparisons. The NSAID association did not remain significant after FDR correction (*q* = 0.142). Logistic regression; outcome  =  receipt of any operative repair. The previously reported Lap-first hospital indicator term was removed: Most hospitals contributed only one patch case in 2018–2022, so that flag was a near-deterministic function of the case’s own procedure (mechanistic association, not informative). The leave-one-out sensitivity model could not be estimated reliably because of sparse data and model separation (Supplementary Appendix S3). Bold values indicate statistical significance (*p* < 0.05). aOR, adjusted odds ratio; CI, confidence interval; NSAID, non-steroidal anti-inflammatory drug.

### 3.8 Center-level practice variation

Hospitals were classified as laparoscopic-first or open-first based on the dominant patch approach for their patch admissions across the study period (Supplementary Table S1). Patients treated at laparoscopic-first centers (356 admissions) had lower mean age (51.2 vs. 54.9 years; *p* < 0.001) and lower mean van Walraven score (2.3 vs. 3.6; *p* < 0.001) compared with patients at open-first centers (159 admissions). Because the case mix at laparoscopic-first centers was younger and healthier, this comparison cannot be interpreted as a causal effect of approach and is presented as a description of practice variation.

## 4 Discussion

### 4.1 Principal findings

In this national, contemporary, ICD-10-era cohort of perforated marginal ulcer after RYGB—the largest assembled to date—non-operative management was the dominant pathway and increased between 2018 and 2022, while laparoscopic patch repair declined. Among operatively managed admissions, open patch repair was associated with longer length of stay and higher rates of sepsis, acute kidney injury, respiratory failure, and venous thromboembolism than laparoscopic patch repair. In a descriptive comparison, patients treated at hospitals where laparoscopic patch was the dominant approach had lower crude mortality than those treated where open patch dominated; however, because case mix differed between center types, this contrast reflects practice variation rather than a treatment effect.

### 4.2 Comparison with prior literature

The 2022 systematic review by Martinino and colleagues pooled approximately 600 patients with perforated marginal ulcer after RYGB across roughly two dozen reports (6). Our cohort nearly triples that pooled estimate, applies survey weights for national projections, and provides the first ICD-10-era contemporary trend description. Prior database studies in this space have addressed marginal ulcer more broadly: MBSAQIP analyses quantified early marginal ulceration after RYGB (14, 15), and an ICD-9-era National Inpatient Sample analysis examined Helicobacter pylori and marginal ulceration after bariatric surgery (16); to our knowledge, no previous NIS, NRD, MBSAQIP, or NSQIP study has specifically examined perforated marginal ulcer after RYGB, its operative management, and its inpatient outcomes. The mortality we observed (approximately 1%) is consistent with reports from individual high-volume centers (17–19). The favorable complication profile of laparoscopic patch compared with open patch echoes the broader perforated peptic ulcer literature, including the LAMA randomized trial (25) and a contemporary multicenter study showing reduced complications and shorter length of stay with laparoscopic omental patch in non-bariatric patients (26). Single-center bariatric series have suggested that revisional gastrojejunostomy or partial reversal can be performed safely in selected patients, particularly for refractory or recurrent disease (18, 19).

### 4.3 Operative approach and surgical decision-making

Our findings are consistent with a laparoscopic-first strategy for patients with perforated marginal ulcer after RYGB whose hemodynamics and intra-abdominal anatomy permit a minimally invasive approach, although the observational design means they cannot establish the superiority of one approach over another. Patients selected for open repair may have had greater physiologic severity, more hostile intra-abdominal anatomy, or more extensive contamination, and confounding by indication of this kind cannot be fully removed by adjustment for administrative covariates. The 41% longer length of stay and FDR-adjusted excess of major complications after open patch repair are concordant with mechanistic expectations: Open approaches in patients with prior bariatric surgery require longer incisions through obese abdominal walls, are associated with greater third-space fluid shifts, and impair early mobilization (26, 27).

### 4.4 The rise of non-operative management

The increase in non-operative management from 64.1% in 2018 to 72.0% in 2022 mirrors broader trends in perforated peptic ulcer care, where selective non-operative management has been advocated for hemodynamically stable patients with sealed perforations (28, 29). Administrative data could not distinguish deliberate non-operative management from admissions involving delayed transfer, comfort-focused care, or endoscopic treatment not captured by the coding algorithm. Prospective triage criteria are needed to separate appropriate non-operative candidates from patients who would benefit from earlier source control.

### 4.5 Modifiable risk factors

Smoking, NSAID exposure, and *Helicobacter pylori* infection are the modifiable risk factors most consistently linked to marginal ulcer in the broader literature (8–10, 12). In our cohort, the marginal association before FDR correction of NSAID exposure with lower receipt of operative repair (raw *p* = 0.045) did not survive false-discovery-rate adjustment, and neither smoking, *Helicobacter pylori* coding, nor obesity diagnosis was associated with operative receipt. We interpret these null results as consistent with the well-known under-coding of behavioral exposures in administrative claims data rather than as evidence against the established epidemiology. Rigorous Helicobacter screening, perioperative smoking cessation, NSAID avoidance, and adequate proton pump inhibitor absorption [which is impaired by RYGB anatomy (13)] remain evidence-based pillars of marginal ulcer prevention (4, 9, 10, 12).

### 4.6 Center-level practice variation

Differences in case mix between center types limit interpretation of their outcome comparisons. Patients at laparoscopic-first centers were younger and had lower comorbidity burden, and these centers had a lower overall operative rate, suggesting that case selection drives a meaningful share of the association. Nonetheless, the volume–outcome literature in foregut and bariatric surgery (30, 31) supports the hypothesis that minimally invasive expertise, postbypass anatomic familiarity, and structured perioperative pathways aggregate at experienced bariatric centers and may benefit patients presenting with perforated marginal ulcer. This center-level comparison remains descriptive, and it should not be interpreted as implying that one type of center provides superior care.

### 4.7 Limitations

Our analysis has several limitations. First, the National Inpatient Sample captures administrative coding rather than chart-level clinical detail; physiologic severity, intraoperative findings, hemodynamics, perforation size, and time to source control cannot be ascertained. Second, behavioral exposures (smoking, non-steroidal anti-inflammatory drug use, and *Helicobacter pylori*) are well known to be under-coded, which may have affected the observed associations. Third, the database reports only in-hospital mortality, missing 30-day, 90-day, and longer-term events. Fourth, with only 19 in-hospital deaths, the events-per-variable ratio precluded a stable multivariable mortality model, and the multivariable contrasts for length of stay and charges should be interpreted as associations rather than causal effects despite cluster-robust standard errors and prespecified sensitivity analyses (24). In particular, patients selected for open repair may have differed systematically in physiologic severity and technical complexity from those approached laparoscopically, so the laparoscopic-vs.-open contrasts remain vulnerable to confounding by indication. Fifth, procedure code grouping into laparoscopic vs. open is imperfect; some operations coded as percutaneous endoscopic may represent hand-assisted laparoscopic procedures, and vice versa. Finally, the case-finding strategy based on perforated gastrojejunal ulcer plus a status code for prior bariatric surgery may miss a small number of perforations captured only through postprocedural complication codes; a sensitivity cohort that additionally included those admissions yielded directionally consistent results.

## 5 Conclusions

In this first national, contemporary analysis of perforated marginal ulcer after Roux-en-Y gastric bypass, non-operative management was the majority pathway and increased between 2018 and 2022, while laparoscopic patch repair declined. When operative repair was performed, a laparoscopic patch approach was associated with shorter length of stay and a more favorable in-hospital complication profile than open patch repair. Practice varied substantially across centers. Because operative approach was not randomized and patients selected for open repair may have been physiologically sicker or anatomically more complex, these findings should not be interpreted as establishing the superiority of a laparoscopic-first strategy. Prospective multicenter studies are needed to refine triage between operative and non-operative pathways and to define which patients are best served by a laparoscopic-first strategy.

## Data Availability

The data were obtained from the HCUP National Inpatient Sample and are available through the HCUP Central Distributor subject to its access requirements and Data Use Agreement. The authors cannot redistribute these data; https://www.hcup-us.ahrq.gov.
