# Supplementary material for: Perforated marginal ulcer after Roux-en-Y gastric bypass: a national analysis of operative approaches, outcomes, and practice variations, 2018–2022
Source: Front Surg. 2026 Oct 21;13:1907935. doi: 10.3389/fsurg.2026.1907935 (PMC13731246; doi:10.3389/fsurg.2026.1907935)
Supplement: Supplementary file 1 [file Supplementaryfile1.docx]

**Table S1. Comparison of admissions treated at laparoscopic-first versus open-first hospitals.**

| **Variable** | **Laparoscopic-first centers (n = 356; wN = 1,780)** | **Open-first centers (n = 159; wN = 795)** | **P-value** | **SMD** |
| --- | --- | --- | --- | --- |
| **DEMOGRAPHICS** | | | | |
| Age, years, mean (SD) | 51.2 (13.0) | 54.9 (12.1) | <0.001 | 0.291 |
| Female, n (%) | 293 (82.3) | 135 (84.9) | 0.116 | 0.07 |
| **BEHAVIORAL** | | | | |
| Smoking, n (%) | 96 (27.0) | 48 (30.2) | 0.102 | 0.071 |
| NSAID exposure, n (%) | Suppressed | Suppressed | Suppressed | Suppressed |
| *H. pylori*, n (%) | Suppressed | Suppressed | Suppressed | Suppressed |
| **COMORBIDITIES** | | | | |
| Van Walraven score, mean (SD) | 2.3 (7.2) | 3.6 (7.4) | <0.001 | 0.172 |
| **PROCEDURES** | | | | |
| Operative repair, n (%) | 231 (64.9) | 123 (77.4) | <0.001 | 0.278 |
| Laparoscopic patch repair, n (%) | 210 (59.0) | Suppressed | Suppressed | Suppressed |
| Open patch repair, n (%) | Suppressed | 116 (73.0) | Suppressed | Suppressed |
| **OUTCOMES** | | | | |
| In-hospital mortality, n (%) | Suppressed | Suppressed | Suppressed | Suppressed |

Centers were classified as laparoscopic-first if the weighted laparoscopic-patch share among patch repairs was ≥50%. Denominators include all admissions at laparoscopic-first or open-first centers, including operative and non-operative admissions, rather than patch cases alone.

Suppressed: counts of 10 or fewer admissions and their corresponding percentages are withheld to protect confidentiality per the HCUP cell suppression policy. Comparison statistics for affected rows are also withheld.

NSAID, non-steroidal anti-inflammatory drug; SD, standard deviation; SMD, standardized mean difference; wN, weighted number of admissions.
