# Supplementary material for: Perforated marginal ulcer after Roux-en-Y gastric bypass: a national analysis of operative approaches, outcomes, and practice variations, 2018–2022
Source: Front Surg. 2026 Oct 21;13:1907935. doi: 10.3389/fsurg.2026.1907935 (PMC13731246; doi:10.3389/fsurg.2026.1907935)
Supplement: Supplementary file 2 [file Supplementaryfile2.docx]

Supplementary Appendix S2. Coding definitions used in the analysis

This appendix documents the implemented ascertainment rules. Diagnostic prefixes were searched across all available diagnosis fields and procedure prefixes across all available procedure fields. A listed prefix includes its matching descendants. These administrative proxies do not establish clinical details that are absent from the underlying codes.

# Cohort and exclusions

**Gastrojejunal ulcer with perforation:** K28.1, K28.2, K28.5, K28.6. These categories include acute or chronic/unspecified gastrojejunal ulcer with perforation, with or without hemorrhage.

**Prior bariatric surgery:** Z98.84. This indicates bariatric-surgery status and does not specifically establish Roux-en-Y gastric bypass.

**Eligibility:** Adults aged 18 years or older with the ulcer and bariatric-status criteria above. Admissions with pregnancy-related diagnoses (O) or selected abdominal, lower-back, pelvic, and external-genital trauma diagnoses (S30, S31, S35, S36, S37, S38, S39) were excluded. A positive, nonmissing discharge weight and nonmissing in-hospital mortality status were required.

**Expanded sensitivity ascertainment:** The additional diagnostic family was K91, postprocedural disorders of the digestive system. This is a broad family and is not specific to perforation or leakage.

# Procedure classification

**Repair procedures:** The implemented repair prefixes were 0DQ6 (stomach) and 0DQA (jejunum). Open and percutaneous endoscopic approaches were identified by the fifth procedure-code character, 0 and 4, respectively. The any-repair flag also accepted the percutaneous approach (character 3). These repair prefixes do not specifically establish use of an omental patch.

**Revisional-category proxy:** The implemented prefixes were 0DW6, 0D16, 0DN6, 0DN8, 0DNA. They encompass revision of a device in the stomach, bypass of the stomach, and release of the stomach, small intestine, or jejunum. These procedures do not individually establish gastrojejunostomy revision or gastric bypass reversal. Only open, percutaneous, and percutaneous endoscopic approaches (fifth character 0, 3, or 4) were included.

**Diagnostic inspection:** The implemented prefix was 0WJG with percutaneous endoscopic approach character 4. An admission with this flag alone was not assigned to an operative treatment group.

**Assignment hierarchy:** The revisional-category proxy took precedence over repair. Remaining repair admissions with an open code were assigned to open repair; those without an open code but with a percutaneous endoscopic repair code were assigned to laparoscopic repair. All other admissions were assigned to the residual non-operative category. Thus, that category means no qualifying procedure under this algorithm, not verified absence of therapeutic intervention.

**Important coverage limitation:** Supplementation of the stomach or jejunum (0DU6 and 0DUA) was not part of the implemented repair definition. The algorithm therefore should not be described as complete ascertainment of all patch repairs.

# Outcomes

**In-hospital mortality:** Recorded death during the hospitalization.

**Length of stay and charges:** Length of stay in days and total hospital charges were taken from the discharge record. Negative sentinel values were treated as missing. The regression analysis adjusted charges to 2022 US dollars.

**Sepsis:** A40, A41, R65.20, R65.21.

**Acute kidney injury:** N17.0, N17.1, N17.2, N17.8, N17.9, N17.

**Respiratory failure:** J96.0, J96.2, J96.1. Includes acute, acute-on-chronic, and chronic respiratory failure.

**Venous thromboembolism:** I26, I82.4.

**Postprocedural infection:** T81.4.

**Persistent postprocedural fistula proxy:** T81.83. This is not an anatomically specific definition of anastomotic leakage.

**Red blood cell transfusion:** 30233N1, 30243N1.

**Postprocedural digestive-system disorders:** K91. The entire K91 family was used; this is broader than postprocedural perforation or leakage.

Diagnosis-based outcomes were identified anywhere in the admission record. The algorithm did not restrict them to conditions arising after admission or after a procedure. Transfusion was identified with procedure codes, not diagnosis codes.

# Behavioral exposures and other covariates

**Smoking:** F17, Z72.0.

**NSAID exposure:** T39.3X5, Z79.1.

**Helicobacter pylori infection:** B96.81.

**Obesity or body-mass-index coding:** E66, Z68.

**Substance use:** F10, F11, F12, F13, F14, F15, F16, F18, F19.

Additional covariates were age, sex, race/ethnicity, expected primary payer, neighborhood income quartile, weekend and elective admission, calendar year, hospital region, and hospital location/teaching status. Calendar year was centered at 2020 for regression. Hospital characteristics were linked within the same calendar year.

# Implemented comorbidity groups and score weights

Each group was flagged if any listed diagnosis prefix matched. The comorbidity count was the sum of these group flags; the weighted score was the sum of the flags multiplied by the weights below. No additional mutual-exclusion hierarchy was applied by the implementation. The lists below reproduce the code as used; they are not a claim of equivalence to a separately validated comorbidity implementation.

**Congestive heart failure:** Score weight 7; diagnosis prefixes: I09.9, I11.0, I13.0, I13.2, I25.5, I42.0, I42.5, I42.6, I42.7, I42.8, I42.9, I43, I50, P29.0.

**Cardiac arrhythmias:** Score weight 5; diagnosis prefixes: I44.1, I44.2, I44.3, I45.6, I45.9, I47, I48, I49, R00.0, R00.1, R00.8, T82.1, Z45.0, Z95.0.

**Valvular disease:** Score weight -1; diagnosis prefixes: A52.0, I05, I06, I07, I08, I09.1, I09.8, I34, I35, I36, I37, I38, I39, Q23.0, Q23.1, Q23.2, Q23.3, Z95.2, Z95.3, Z95.4.

**Pulmonary circulation disorders:** Score weight 4; diagnosis prefixes: I26, I27, I28.0, I28.8, I28.9.

**Peripheral vascular disorders:** Score weight 2; diagnosis prefixes: I70, I71, I73.1, I73.8, I73.9, I77.1, I79.0, I79.2, K55.1, K55.8, K55.9, Z95.8, Z95.9.

**Uncomplicated hypertension:** Score weight 0; diagnosis prefixes: I10.

**Complicated hypertension:** Score weight 0; diagnosis prefixes: I11, I12, I13, I15.

**Paralysis:** Score weight 7; diagnosis prefixes: G04.1, G11.4, G80.1, G80.2, G81, G82, G83.0, G83.1, G83.2, G83.3, G83.4, G83.9.

**Other neurological disorders:** Score weight 6; diagnosis prefixes: G10, G11, G12, G13, G20, G21, G22, G25.4, G25.5, G31.2, G31.8, G31.9, G32, G35, G36, G37, G40, G41, G93.1, G93.4, R47.0, R56.

**Chronic pulmonary:** Score weight 3; diagnosis prefixes: I27.8, I27.9, J40, J41, J42, J43, J44, J45, J46, J47, J60, J61, J62, J63, J64, J65, J66, J67, J68.4, J70.1, J70.3.

**Diabetes uncomplicated:** Score weight 0; diagnosis prefixes: E10.0, E10.1, E10.9, E11.0, E11.1, E11.9, E12.0, E12.1, E12.9, E13.0, E13.1, E13.9, E14.0, E14.1, E14.9.

**Diabetes complicated:** Score weight 0; diagnosis prefixes: E10.2, E10.3, E10.4, E10.5, E10.6, E10.7, E10.8, E11.2, E11.3, E11.4, E11.5, E11.6, E11.7, E11.8, E12.2, E12.3, E12.4, E12.5, E12.6, E12.7, E12.8, E13.2, E13.3, E13.4, E13.5, E13.6, E13.7, E13.8, E14.2, E14.3, E14.4, E14.5, E14.6, E14.7, E14.8.

**Hypothyroidism:** Score weight 0; diagnosis prefixes: E00, E01, E02, E03, E89.0.

**Renal failure:** Score weight 5; diagnosis prefixes: I12.0, I13.1, N18, N19, N25.0, Z49.0, Z49.1, Z49.2, Z94.0, Z99.2.

**Liver disease:** Score weight 11; diagnosis prefixes: B18, I85, I86.4, I98.2, K70, K71.1, K71.3, K71.4, K71.5, K71.7, K72, K73, K74, K76.0, K76.2, K76.3, K76.4, K76.5, K76.6, K76.7, K76.8, K76.9, Z94.4.

**Peptic ulcer disease:** Score weight 0; diagnosis prefixes: K25, K26, K27, K28.

**HIV/AIDS:** Score weight 0; diagnosis prefixes: B20.

**Lymphoma:** Score weight 9; diagnosis prefixes: C81, C82, C83, C84, C85, C88, C96, C90.0, C90.2.

**Metastatic cancer:** Score weight 12; diagnosis prefixes: C77, C78, C79, C80.

**Solid tumors:** Score weight 4; diagnosis prefixes: C00, C01, C02, C03, C04, C05, C06, C07, C08, C09, C10, C11, C12, C13, C14, C15, C16, C17, C18, C19, C20, C21, C22, C23, C24, C25, C26, C30, C31, C32, C33, C34, C37, C38, C39, C40, C41, C43, C45, C46, C47, C48, C49, C50, C51, C52, C53, C54, C55, C56, C57, C58, C60, C61, C62, C63, C64, C65, C66, C67, C68, C69, C70, C71, C72, C73, C74, C75, C76, C97.

**Rheumatic and connective-tissue disorders:** Score weight 0; diagnosis prefixes: L94.0, L94.1, L94.3, M05, M06, M08, M12.0, M12.3, M30, M31.0, M31.1, M31.2, M31.3, M32, M33, M34, M35, M45, M46.1, M46.8, M46.9.

**Coagulopathy:** Score weight 3; diagnosis prefixes: D65, D66, D67, D68, D69.1, D69.3, D69.4, D69.5, D69.6.

**Obesity:** Score weight -4; diagnosis prefixes: E66.

**Weight loss:** Score weight 6; diagnosis prefixes: E40, E41, E42, E43, E44, E45, E46, R63.4, R64.

**Fluid electrolyte:** Score weight 5; diagnosis prefixes: E22.2, E86, E87.

**Blood loss anemia:** Score weight -2; diagnosis prefixes: D50.0.

**Deficiency anemia:** Score weight -2; diagnosis prefixes: D50.8, D50.9, D51, D52, D53.

**Alcohol-related disorders:** Score weight 0; diagnosis prefixes: F10, E52, G62.1, I42.6, K29.2, K70.0, K70.3, K70.9, T51, Z50.2, Z71.4, Z72.1.

**Drug use disorders:** Score weight -7; diagnosis prefixes: F11, F12, F13, F14, F15, F16, F18, F19, Z71.5, Z72.2.

**Psychoses:** Score weight 0; diagnosis prefixes: F20, F22, F23, F24, F25, F28, F29, F30.2, F31.2, F31.5.

**Depression:** Score weight -3; diagnosis prefixes: F20.4, F31.3, F31.4, F31.5, F32, F33, F34.1, F41.2, F43.2.

# Definition references

https://www.cms.gov/icd10m/version35-fullcode-cms/fullcode_cms/P0978.html

https://www.cms.gov/icd10m/version36-fullcode-cms/fullcode_cms/P1024.html

https://www.cms.gov/icd10m/FY2025-NPRM-Version42-fullcode-cms/fullcode_cms/P1057.html

https://www.cms.gov/icd10m/FY2025-NPRM-Version42-fullcode-cms/fullcode_cms/P0895.html

https://www.cms.gov/icd10m/FY2026-fr-v43-fullcode-cms/fullcode_cms/P1426.html

https://www.cms.gov/icd10m/fy2025-version42.1-fullcode-cms/fullcode_cms/P2158.html

The cited manuals clarify the specified code meanings. This appendix does not establish annual completeness or validity of every prefix across all study years.
