# Supplementary material for: Perforated marginal ulcer after Roux-en-Y gastric bypass: a national analysis of operative approaches, outcomes, and practice variations, 2018–2022
Source: Front Surg. 2026 Oct 21;13:1907935. doi: 10.3389/fsurg.2026.1907935 (PMC13731246; doi:10.3389/fsurg.2026.1907935)
Supplement: Supplementary file 3 [file Supplementaryfile3.docx]

Supplementary Appendix S3. Leave-one-out sensitivity analysis

# Purpose and definition

This sensitivity analysis assessed whether the operative approach used for other patch-repair admissions at the same hospital was associated with receipt of operative repair. For each admission, hospital preference was calculated from the discharge-weighted proportion of laparoscopic repair among other patch-repair admissions in the same calendar year. A proportion of at least 50% defined laparoscopic-first practice.

Hospitals were identified separately within each calendar year because National Inpatient Sample hospital identifiers are reassigned annually. The index admission was excluded from its own hospital-preference calculation. When no other patch-repair admission was available, hospital preference was treated as unavailable rather than classified as open-first.

# Model assessment

Among 1,669 cohort admissions, the leave-one-out classification was available for 151; 53 of these admissions underwent operative repair. The model included smoking, NSAID exposure, Helicobacter pylori infection, obesity or body-mass-index coding, substance use, age, sex, comorbidity score, calendar year, elective admission, and leave-one-out hospital preference. Discharge weights and standard errors clustered by hospital within calendar year were used, retaining the original covariate specification.

The model exhibited separation and had fewer than five operative events per predictor. In accordance with the analysis stability threshold, adjusted odds ratios, confidence intervals, and p-values from this model were not reported. The data did not support a stable estimate of the association between leave-one-out hospital preference and operative repair.

# Interpretation

This sensitivity analysis is inconclusive and does not establish either the presence or absence of a hospital-preference association. It retains the original treatment-classification algorithm and therefore does not resolve its coding limitations. No replacement effect estimate is proposed.

Hospital-identifier documentation: https://hcup-us.ahrq.gov/db/nation/nis/nisdde.jsp
